# Zinc supplementation strategies in feedlot heifers receiving an extended-release implant or an aggressive re-implant program

**DOI:** 10.1093/jas/skab212

**Published:** 2021-07-14

**Authors:** Elizabeth M Messersmith, Emma K Niedermayer, Kara J Thornton, Grant I Crawford, Stephanie L Hansen

**Affiliations:** 1 Department of Animal Science, Iowa State University, Ames, IA 50014, USA; 2 Department of Animal, Dairy, and Veterinary Science, Utah State University, Logan, UT 84322, USA; 3 Merck Animal Health, Madison, NJ 07940, USA

**Keywords:** anabolic implant, beef cattle, extended-release, muscle fiber cross-sectional diameter, trace mineral

## Abstract

Two hundred eight Angus-crossbred heifers (291 ± 23 kg) from four sources were used in a randomized complete block design. The objective of the study was to determine the effects of implant strategy and Zn supplementation on performance, carcass characteristics, muscle fiber diameter, and mineral status of heifers. Heifers were assigned to a 2 × 2 factorial study for 168 d, and factors included Zn and implant (IMP). Heifers were supplemented Zn (mg/kg dry matter [DM]; ZnSO_4_) at national (30; NRC) or industry (100; IND) recommendations. Implant strategies (Merck Animal Health, Madison, NJ) included extended-release Revalor-XH on day 0 (REV-XH; 20 mg estradiol + 200 mg trenbolone acetate) containing four uncoated pellets and six coated pellets or the uncoated implant Revalor-200 on day 0 and again on day 91 (REV-200; 20 mg estradiol + 200 mg trenbolone acetate). Heifers were blocked by weight within source to pens of five or six heifers per pen (nine pens per treatment). A corn silage-based diet was fed during the growing period (days 0–55) followed by transition to a corn-based finishing diet. Weights were taken consecutively on days −1/0, 55/56, and 167/168. Liver and muscle from the longissimus thoracis were collected from one heifer per pen on days −5, 14, 105, and 164. Data were analyzed via Mixed Procedure of SAS. Average daily gain (ADG) and liver mineral used Period as the repeated effect. Corresponding to periods of high hormone payout from each implant, days 0–28 and 91–120 ADG were greatest for REV-200, whereas REV-XH numerically peaked during days 56–91 (IMP × Period; *P* = 0.02). Day 91 IND body weight tended to be heavier (*P* = 0.06) and day 120 body weight was heavier (*P* = 0.05) than NRC heifers. No effect of Zn or IMP on final body weight was observed (*P* ≥ 0.21). Muscle fiber cross-sectional diameter on day 164 was greater (*P* = 0.05) in IND than NRC. Liver Mn concentrations decreased by day 14 regardless of implant, though days 105 and 164 concentrations were lesser for REV-200 than REV-XH (IMP × Period; *P* = 0.02). No effects of Zn, IMP, or the interaction were observed for carcass-adjusted gain to feed, days 0–168 DM intake, hot carcass weight, or ribeye area (*P* ≥ 0.11). The nominal differences in performance between implant strategies suggest that extended-release implants may be an effective implant strategy to replace re-implant programs in heifers, whereas the improved performance of heifers fed IND vs. NRC during times of peak hormone payout suggests a role for Zn in periods of rapid growth.

## Introduction

The use of anabolic steroidal implants in the feedlot is a common practice (Samuelson et al., 2016), with approximately 84% of cattle on feed in the United States receiving an implant upon arrival and 71% of cattle receiving an implant at a second processing date (NAHMS, 2013). Continuous anabolic implant exposure through an initial and terminal implant program improves growth performance (Reinhardt, 2007), but additional costs are associated with a second processing date. Revalor-XH, an extended-release implant developed specifically for heifers, provides 200 mg trenbolone acetate + 20 mg estradiol (Merck Animal Health; Madison, NJ) evenly dispersed across 10 pellets (FDA [Food and Drug Administration], 2017a). Revalor-XH consists of four uncoated and six coated pellets (FDA, 2017a). Uncoated pellets provide immediate release of trenbolone acetate and estradiol, whereas hormone payout from coated pellets begins at approximately day 70 (FDA, 2017b). In a re-implant program, feedlot heifers may receive Revalor-200 (200 mg trenbolone acetate + 20 mg estradiol; Merck Animal Health) as both the initial and terminal implants. This strategy offers more than twice the anabolic hormone dose in comparison to a single Revalor-XH. Although increased hormone potency may be expected to improve cattle growth (Bartle et al., 1992), previous studies have reported minimal differences in final body weight (BW) between these strategies (Crawford et al., 2018; Ohnoutka et al., 2018). However, few studies have reported cattle performance during interim periods when comparing extended-release implant and re-implant programs.

Zinc may be essential to support optimal carcass accretion in implanted cattle as lambs implanted with zeranol exhibited greater absorption and retention of Zn (Hufstedler and Greene, 1995). Furthermore, Carmichael et al. (2018) reported a positive correlation between Zn and N retention in late-stage finishing beef steers. Arguably, the most utilized trace mineral in the body, Zn is involved in thousands of proteins (Andreini et al., 2006) and has a vital role in protein synthesis (Wegener and Romano, 1963; Oberleas and Prasad, 1969). In spite of substantial improvements in beef cattle growth due to genetic selection and development of growth promoting technologies, Zn recommendations have remained steady (NRC, 1996; NASEM, 2016), though nutritional consultants report feeding Zn at three times national recommendations (Samuelson et al., 2016). Niedermayer et al. (2018) found increasing supplemental trace mineral concentrations to industry rather than national recommendations improved hot carcass weight (HCW) and feed efficiency in steers. Therefore, the objective of this study was to determine if supplemental Zn (as ZnSO_4_) at national (30 mg Zn/kg dry matter [DM]) or industry (100 mg Zn/kg DM) recommendations would affect the performance of heifers given a two-implant (Revalor-200, Revalor-200) or extended-release (Revalor-XH) implant strategy. It was hypothesized that increased supplemental Zn would increase the growth rate with greatest effects in heifers receiving a two-implant strategy compared to a single extended-release implant.

## Materials and Methods

The Iowa State University Institutional Animal Care and Use Committee (log number: IACUC-18-103) approved all procedures and protocols used in this study.

### Animals and experimental design

A total of 208 Angus-crossbred heifers (291 ± 23 kg) from four sources of origin were used in a 2 × 2 factorial randomized complete block design study examining the effect of Zn supplementation on the performance of beef heifers receiving different implant strategies throughout the 168-d study conducted from July through December 2018. Cattle were processed before the start of the study to induce luteolysis (Estrumate; Merck Animal Health, Madison, NJ), prevent clostridial diseases (Vision 7; Merck Animal Health), deter respiratory diseases (Vista Once; Merck Animal Health), and defend against parasites (Safe-Guard; Merck Animal Health). Within the source, heifers were blocked by weight to pens of 5 or 6 (36 pens total) and fed a corn silage-based growing diet from days 0–56 and then transitioned to a dry rolled corn-based finishing diet for the remainder of the study (Table 1). Each block consisted of four pens (*n* = 1 per treatment). Melengestrol acetate (MGA), rumensin, and trace minerals were supplemented to the diet through dried distillers grains with soluble-based premixes. During the first 23 d of the growing diet an ingredient DM error occurred, though differences in MGA, rumensin, and Zn supplementation were negligible. The error was corrected on day 24 and is reflected in the diet table. Cattle were fed ad libitum via concrete bunks at approximately 0800 h daily and had access to automatic waterers. Two dietary Zn treatments were fed, including Zn supplementation at national (NRC; 30 mg Zn/kg DM; NASEM, 2016) or industry (IND; 100 mg Zn/kg DM; Samuelson et al., 2016) recommendations from ZnSO_4_. Within Zn treatment, heifers received either the extended-release implant Revalor-XH (200 mg trenbolone acetate + 20 mg estradiol; Merck Animal Health) on day 0 or Revalor-200 (200 mg trenbolone acetate + 20 mg estradiol; Merck Animal Health) on day 0 and again on day 91. There were nine pens per full factorial treatment combination. Cattle were shipped to a commercial abattoir (Iowa Premium Beef, Tama, IA; trucking distance: 100 km) in the afternoon of day 168 and were harvested the morning of day 169.

**Table 1.** Diet composition

% DM basis	Growing^1^,^2^	Finishing^3^
Ingredient		
Dry rolled corn	—	40.0
Sweet Bran^4^	40.0	20.0
Corn silage	40.0	20.0
DDGS^5^	18.04	18.04
Limestone	1.5	1.5
Salt	0.31	0.31
Vitamin premix^6^	0.1	0.1
Mineral premix^7^	0.02	0.02
Rumensin^8^	0.0135	0.0135
MGA^9^	0.0134	0.0134
Analyzed composition^10^		
Crude protein^10^	18.44	15.45
NDF^10^	33.02	22.46
Ether extract^10^	4.14	4.32
NEg, Mcal/kg^11^	1.30	1.40
Sulfur^10^	0.31	0.25
Cu, mg/kg DM^12^	15	14
Fe, mg/kg DM^12^	90	69
Mn, mg/kg DM^12^	40	38
Zn, mg/kg DM^12^,^13^	77	68

^1^Growing period days 0–55; heifers transitioned to finishing diet over two transition diets.

^2^Ingredient dry matter error occurred during the first 23 d of growing period before correction to displayed growing diet. Differences in MGA, rumensin, and Zn supplementation were negligible.

^3^Finishing period days 84–169.

^4^Branded wet corn gluten feed (Cargill Corn Milling, Blair, NE).

^5^Dried distillers grains with solubles.

^6^Premix provided 2,200 IU vitamin A and 25 IU vitamin E/kg diet.

^7^Minerals were provided at NASEM (2016) recommendations for Co, Mn, Se, Cu, and I from inorganic sources in addition to 30 (NRC) or 100 (IND) mg Zn/kg DM from ZnSO_4_.

^8^Active ingredient Monensin (Elanco, Greenfield, IN).

^9^Melengestrol acetate (Zoetis, Florham Park, NJ).

^10^Analysis of total mixed rations by Dairyland Laboratories (Arcadia, WI).

^11^Net energy of gain was calculated using NASEM (2016) values for ingredients.

^12^Analyzed values for trace minerals represent NRC treatment for both growing and finishing periods measured by inductively coupled plasma optical emission spectrometry (ICP Optima 7000 DV, Perkin Elmer, Waltham, MA).

^13^Analyzed Zn concentrations for IND treatment total mixed ration during growing and finishing periods were 149 and 129 mg Zn/kg DM, respectively.

### Sample collection and analysis

All heifers were weighed individually on consecutive days at the beginning of the study (day −1/0), end of the growing period (days 55/56), and end of the study (days 167/168). Intermediate BW was collected on days 28, 91, and 120. Cattle were harvested on day 169 at a commercial abattoir and carcass characteristics were collected by trained University personnel on the left half of the carcass. No camera data were available. Hot carcass weight data were recorded at harvest and following a 48-h chill, ribeye area (REA), back fat (BF), kidney, pelvic, heart fat (KPH), and marbling score were collected, and yield grade (YG) was calculated. Carcass-adjusted final BW was calculated by dividing HCW by treatment averages for dressing percent (63.46%, 63.95%, 64.14%, and 63.90% for NRC/REV-XH, NRC/REV-200, IND/REV-XH, and IND/REV-200, respectively) and gain to feed ratio (G:F) was calculated by dividing average daily gain (ADG) by dry matter intake (DMI).

Liver and muscle biopsies (*n* = 9 per treatment) were conducted on one heifer, randomly selected prior to initiation of the study, from each pen on days −5, 14, 105, and 164 following the method described by Engle and Spears (2000) and adapted procedures from Pampusch et al. (2008), respectively. These sampling days represented initial and final samples as well as 14 d post-implant administration to capture the effects of peak hormonal payout of implants on liver and muscle parameters. Muscle samples were collected from the *longissimus thoracis* between the 10th and 13th ribs. Rib space and side of biopsy were alternated between time points. Liver and composites of total mixed ration (TMR) were acid digested with trace mineral grade nitric acid (Fisher Scientific, Fair Lawn, NJ), following the procedures outlined by Richter et al. (2012). Inductively coupled plasma optical emission spectrometry (Optima 7000 DV, Perkin Elmer, Waltham, MA) was used to analyze plasma, liver, and TMR composites for trace mineral concentration as described by Pogge and Hansen (2013) and Richter et al. (2012).

Muscle samples were analyzed for cross-sectional diameter and area at Utah State University. Samples from the *longissimus thoracis* were mounted with the muscle fibers perpendicular to the cork, cryosectioned (10 μm thick), and used for histochemical fiber-type staining. Serially sectioned samples were stained using a succinate dehydrogenase stain following previously described methods (Pearse, 1968; Picard et al., 1998; Thornton et al., 2012). In brief, samples were incubated for 60 min in a medium consisting of 0.2 M phosphate buffer, 0.17 M sodium succinate, and 1.2 mM nitro blue toluene. Then, samples were washed serially with deionized H_2_O, 30% acetone, 60% acetone, 90% acetone, 60% acetone, 30% acetone, and deionized water, respectfully. Cover slips were then mounted onto sectioned samples using a mounting medium containing 0.09 M gelatin, 6.7 M glycerol, and 0.12 M phenol. Images of sectioned samples were taken using a Zeiss AXIO Observer.Z1 microscope (Carl Zeiss Meditec, Inc., Dublin, CA). At least three different captured images from each sample were taken and used for analysis. Using Image-Pro Plus (Media Cybernetics, Inc., Rockville, MD), average area and diameter were calculated from at least 20 skeletal muscle fibers of each sample.

Weekly TMR samples were dried in a forced air oven at 70 °C for 48 h for determination of diet DM. Dried TMR were ground through a 2-mm screen (Retsch Zm100 grinder; Glen Mills Inc., Clifton, NJ) and samples were composited for each Zn treatment by month within growing and finishing period. Excess feed in the bunk was weighed and a sub-sample was dried as previously described at the end of the growing and finishing period for the calculation of feed disappearance during these periods. Total DMI per pen was calculated utilizing DM from weekly TMR. The DM weight of excess feed was subtracted from each pen, and this value was divided by the number of heifers in each pen to determine the average heifer DMI for the pen. After proper weighing and sampling, off condition feed was discarded as necessary throughout the study.

### Statistical analysis

Performance and carcass data of this randomized complete block design were analyzed as a 2 × 2 factorial using the Mixed Procedure of SAS 9.4 (SAS Inst. Inc., Cary, NC). The model included the fixed effects of Zn, implant, the interaction, and block. Initial BW was used as a covariate in the analysis of performance and carcass characteristics. Analysis of ADG and liver trace mineral was conducted as repeated measures with Period or day of sampling as the repeated effect, respectively. Compound symmetry and unstructured covariance matrixes were selected for ADG and trace mineral analysis, respectively, based on the lowest Akaike Information Criterion. The experimental unit was pen (*n* = 9 per treatment). Cook’s *D* was used to assess outliers. Outliers were discovered for muscle cross-sectional diameter (day 1: REV-XH-IND [1], day 2: REV-XH-NRC [2] and REV-200-NRC [1], and day 3: REV-XH-IND [1]), but no other variables. Six heifers, all from different pens, were removed from the study due to health issues unrelated to experimental treatment (REV-XH-NRC: 1, REV-XH-IND: 2, REV-200-NRC: 1, and REV-200-IND: 2). Data for these animals were removed from analysis on the day of dismissal from the study. All data are reported as least square means with the standard error of the mean. Data were determined statistically significant at *P* ≤ 0.05, and a statistical tendency at 0.05 < *P* ≤ 0.10.

## Results

### Performance and carcass characteristics

Performance parameters are displayed in Table 2. Heifer BW was not affected by Zn × IMP (*P* ≥ 0.27), and Zn treatment did not affect initial, day 28, day 56, or final live BW (*P* ≥ 0.34). However, IND heifers tended to be heavier on day 91 (*P* = 0.06) and were heavier on day 120 (*P* = 0.05), with a 7 kg advantage over NRC by day 120. Implant did not affect initial, day 91, or final live BW (*P* ≥ 0.21), but interim BW was affected by IMP, as REV-200 heifers were heavier on days 28 and 56 (*P* ≤ 0.04) and tended to be heavier on day 120 (*P* = 0.09).

**Table 2.** Effect of zinc^1^ supplementation and implant^2^ strategy on the performance of finishing heifers

	NRC		IND		SEM	P-value		
	REV-XH	REV-200	REV-XH	REV-200		Zn	IMP	Zn × IMP
Pens (*n*)	9	9	9	9				
Live performance^3^								
BW, kg								
Day 0 (Initial)	294	295	294	295	7.0	0.96	0.90	0.98
Day 28	337	342	338	343	1.7	0.34	0.004	0.98
Day 56	381	385	382	387	2.3	0.50	0.04	0.86
Day 91 (Re-implant)	439	442	445	447	2.8	0.06	0.35	0.83
Day 120	485	492	493	498	3.3	0.05	0.09	0.86
Day 168	561	571	570	570	4.6	0.37	0.21	0.27
DMI, kg								
Days 0–90	9.5	9.6	9.6	9.6	0.11	0.68	0.51	0.89
Days 91–168	11.4	11.3	11.6	11.7	0.20	0.09	0.97	0.58
Days 0–168	10.4	10.4	10.6	10.6	0.13	0.15	0.80	0.72
G:F								
Days 0–90	0.170	0.172	0.176	0.177	0.0025	0.04	0.44	0.94
Days 91–168^3^	0.137^b^	0.147^a^	0.138^b^	0.135^b^	0.0030	0.06	0.25	0.03
CA performance^4^,^5^								
Final BW, kg	561	572	570	571	5.0	0.45	0.25	0.34
Overall ADG, kg	1.58	1.65	1.64	1.64	0.030	0.47	0.28	0.29
Overall G:F	0.154	0.160	0.156	0.156	0.0021	0.71	0.18	0.11

^1^The NRC treatment received 30 mg supplemental Zn/kg DM from ZnSO_4_ and IND treatment received 100 mg supplemental Zn/kg DM from ZnSO_4_.

^2^Implant strategies included either a single Revalor-XH implant (REV-XH; 20 mg estradiol + 200 mg TBA; Merck Animal Health, Madison, NJ) on day 0 or a Revalor-200 implant on day 0 and again on day 91 (REV-200; 20 mg estradiol + 200 mg TBA; Merck Animal Heath, Madison, NJ).

^3^Unlike superscripts indicate differences in treatment means (*P*≤ 0.05).

^4^Day 0 BW was used as a covariate in performance analysis, excluding day 0 BW.

^5^Carcass-adjusted (CA) overall performance was determined with the average dressing percent of treatment groups (63.46%, 63.95%, 64.14,% and 63.90% for NRC/REV-XH, NRC/REV-200, IND/REV-XH, and IND/REV-200, respectively).

Dry matter intake was unaffected by Zn × IMP (*P* ≥ 0.58) or IMP (*P* ≥ 0.51). Dry matter intake during the initial implant period and days 0–168 DMI was not affected by Zn (*P* ≥ 0.15); however, IND tended to have greater DMI than NRC during the re-implant period (days 91–168; *P* = 0.09). There was no interaction between Zn and IMP for the initial implant period G:F (*P* = 0.94), nor was there an effect of IMP *(P* = 0.44), whereas IND improved feed efficiency during this period (days 0–90; *P* = 0.04). However, re-implant period G:F was affected by Zn × IMP (*P* = 0.03), where feed efficiency was greater for REV-200-NRC heifers than any other treatment (days 91–169). No differences in carcass-adjusted final BW, overall ADG, or overall G:F due to Zn, IMP, or the interaction were observed (*P* ≥ 0.11).

**Figure 1.**
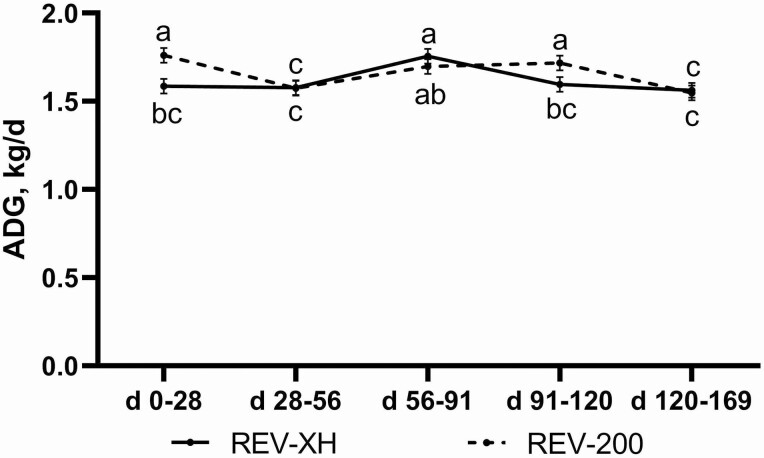
The effect of implant strategy and day on ADG throughout the 169-d study (IMP × Period, *P* = 0.02). Implant treatments included Revalor-XH (REV-XH; 200 mg trenbolone acetate + 20 mg estradiol; Merck Animal Health Madison, NJ) on day 0 or Revalor-200 (REV-200; 200 mg trenbolone acetate + 20 mg estradiol; Merck Animal Health) on day 0 and again on day 91. Data were analyzed using repeated measures of the mixed procedure in SAS. Unlike superscripts indicate differences between means across all time points (*P* ≤ 0.05).

Average daily gain data analyzed as repeated measures across the study indicated an effect of IMP × Period (*P* = 0.02; Figure 1). In general, improvements in ADG followed expected peak hormone payout for the two implant strategies, where ADG was greater from days 0–28 and 91–120 for REV-200 vs. REV-XH, whereas ADG was not different due to IMP during the periods of days 28–56, 56–91, or 120–168. Additionally, no Zn, IMP, Zn × IMP, Zn × Period, or Zn × IMP × Period effects were observed for ADG (*P* ≥ 0.12; Period *P* = 0.0001; Period effect data not shown).

Carcass characteristics shown in Table 3 indicated no Zn, IMP, or Zn × IMP effects for HCW, REA, BF, KPH, marbling, or YG (*P ≥* 0.11). However, dressing percentage tended (*P* = 0.08) to be affected by the interaction of Zn and IMP, where Rev-XH-IND heifers had greater dressing percent than REV-XH-NRC, whereas REV-200 heifers were intermediate, regardless of Zn treatment.

**Table 3.** Effect of zinc^1^ supplementation and implant^2^ strategy on carcass characteristics of finishing beef heifers

	NRC		IND		*SEM*	*P-*value		
	REV-XH	REV-200	REV-XH	REV-200		Zn	IMP	Zn × IMP
Pens (*n*)	9	9	9	9				
Carcass characteristics^3^								
HCW, kg	356	366	365	365	3.2	0.19	0.18	0.11
Dress^4^, %	63.5^y^	64.0^xy^	64.1^x^	63.9^xy^	0.21	0.17	0.59	0.08
REA, sq cm	79.0	82.1	81.3	81.0	1.07	0.60	0.18	0.11
Back fat, cm	1.81	1.94	1.94	1.89	0.073	0.60	0.54	0.24
KPH, %	2.6	2.7	2.7	2.6	0.03	0.82	0.57	0.11
Marbling^5^	576	554	571	548	16.7	0.75	0.18	0.99
YG^6^	3.87	3.94	3.97	3.93	0.110	0.68	0.91	0.64

^1^The NRC treatment received 30 mg Zn/kg DM from ZnSO_4_ and IND treatment received 100 mg Zn/kg DM from ZnSO_4_.

^2^Implant strategies included either a single Revalor-XH implant (REV-XH; 20 mg estradiol + 200 mg TBA; Merck Animal Health, Madison, NJ) on day 0 or a Revalor-200 on day 0 and again on day 91 (REV-200; 20 mg estradiol + 200 mg TBA; Merck Animal Heath, Madison, NJ).

^3^Day 0 BW was used as covariate in the analysis of carcass characteristics.

^4^Unlike superscripts indicate tendencies for differences in treatment means (0.05 < *P* ≤ 0.10).

^5^Marbling scores: slight = 300, small = 400, modest = 500, moderate = 600, slightly abundant = 700, moderately abundant = 800.

^6^Yield grade (YG) was calculated utilizing the USDA yield grade equation.

### Liver and muscle parameters

**Figure 2.**
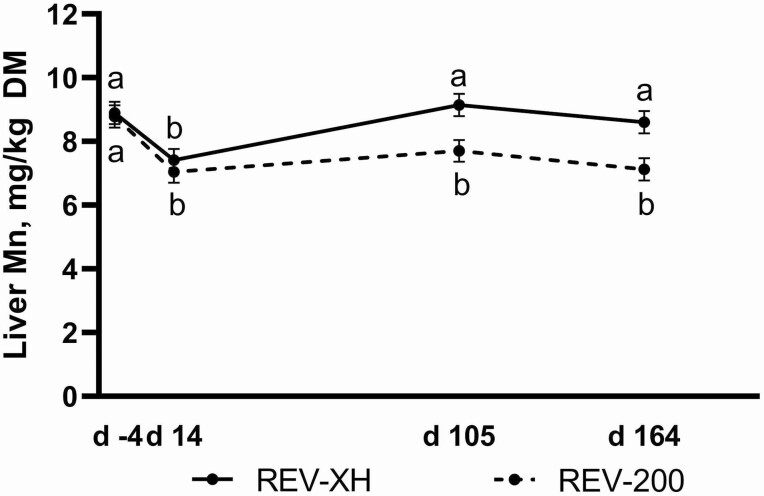
Liver manganese response to implant strategy over course of study (IMP × Day, *P* = 0.02). Heifers received either Revalor-XH (REV-XH; 200 mg trenbolone acetate + 20 mg estradiol; Merck Animal Health; Madison, NJ) on day 0 or Revalor-200 on day 0 and again on day 91 (REV-200; 200 mg trenbolone acetate + 20 mg estradiol; Merck Animal Health). Data were analyzed as repeated measures of the mixed procedure of SAS. Unlike superscripts indicate differences between treatment means across all time points (*P* ≤ 0.05).

Treatment means for liver trace mineral concentrations analyzed as repeated measures are shown in Table 4. Liver Cu, Fe, and Zn concentrations were not affected by Zn, IMP, Zn × IMP, Zn × Day, IMP × Day, or Zn × IMP × Day (*P ≥* 0.12). However, a Zn × IMP effect (*P* = 0.03) was observed for liver Mn concentrations where, within IND heifers, concentrations were less in REV-200 vs. REV-XH. No difference in liver Mn concentrations between implant strategies within NRC treatment was observed. Liver Mn decreased across the first 14 d regardless of implant strategy, but REV-XH heifers returned to initial concentrations by day 105 while REV-200 heifers maintained lesser liver Mn concentrations from day 14 to the end of the study (Figure 2; IMP × Day; *P* = 0.02). No Zn × Day or Zn × IMP × Day effects (*P* ≥ 0.58) were observed for liver Mn concentrations, whereas a Day effect was observed for liver Cu, Fe, Mn, and Zn (*P* ≤ 0.0002).

**Table 4.** Effect of zinc^1^ supplementation and implant^2^ strategy on liver mineral concentrations of finishing heifers

	NRC		IND		SEM	*P-*value		
	REV-XH	REV-200	REV-XH	REV-200		Zn	IMP	Zn × IMP
Heifers (*n*)	9	9	9	9				
Trace mineral concentrations^3^								
Liver, mg/kg DM^4^,^5^								
Cu	350	371	353	321	26.8	0.28	0.85	0.24
Fe	172	167	167	171	9.5	0.97	0.99	0.61
Mn^6^	8.32^a^	7.94^ab^	8.71^a^	7.40^b^	0.217	0.70	0.0007	0.03
Zn	113	117	115	121	4.1	0.47	0.13	0.81

^1^The NRC treatment received 30 mg Zn/kg DM from ZnSO_4_ and IND treatment received 100 mg Zn/kg DM from ZnSO_4_.

^2^Implant strategies included either a single Revalor-XH implant (REV-XH; 20 mg estradiol + 200 mg TBA; Merck Animal Health, Madison, NJ) on day 0 or a Revalor-200 on day 0 and again on day 91 (REV-200; 20 mg estradiol + 200 mg TBA; Merck Animal Heath, Madison, NJ).

^3^Data were analyzed using repeated measures of the mixed procedure of SAS and represent overall treatment means.

^4^No Zn × IMP × Day (*P* ≥ 0.25) effect was observed. Day (*P* ≤ 0.0002); IMP × Day (*P* ≥ 0.12) except for Mn (Figure 2; *P* = 0.02).

^5^Liver samples were collected on days −5, 14, 105, and 164.

^6^Unlike superscripts indicate differences in treatment means (*P*≤ 0.05).

Muscle cross-sectional diameter and area measured from biopsy samples removed from the *longissimus thoracis* at three time points are shown in Table 5. No effects of Zn, IMP, or the interaction were observed on day 14 or 105 (*P* ≥ 0.12) on cross-sectional diameter. Near the end of the study, day 164, muscle cross-sectional diameter was greater for IND supplemented heifers than NRC (*P* = 0.05). However, no IMP or Zn × IMP effects were observed for day 164 muscle cross-sectional diameter (*P* ≥ 0.28) and no Zn, IMP, or Zn × IMP effects were observed for muscle cross-sectional area on day 14, 105, or 164 (*P* ≥ 0.11).

**Table 5.** Effect of zinc^1^ supplementation and implant^2^ strategy on *longissimus thoracis* cross-sectional diameter and area from finishing heifers

	NRC		IND		SEM	*P-*value		
	REV-XH	REV-200	REV-XH	REV-200		Zn	IMP	Zn × IMP
Heifers (*n*)	9	9	9	9				
Muscle cross-sectional diameter, µm								
Day 14	50.0	48.7	53.9	50.4	2.05	0.12	0.19	0.54
Day 105	69.0	68.1	69.7	74.3	3.16	0.23	0.52	0.36
Day 164	61.3	57.5	69.3	64.8	4.28	0.05	0.28	0.93
Muscle cross-sectional area, µm^2^								
Day 14	2,144	2,026	2,350	2,183	175.7	0.22	0.33	0.87
Day 105	4,016	4,000	4,157	4,765	385.6	0.20	0.40	0.40
Day 164	3,250	2,958	3,769	3,674	451.6	0.11	0.61	0.79

^1^The NRC treatment received 30 mg Zn/kg DM from ZnSO_4_ and IND treatment received 100 mg Zn/kg DM from ZnSO_4_.

^2^Implant strategies included either a single Revalor-XH implant (REV-XH; 20 mg estradiol + 200 mg TBA; Merck Animal Health, Madison, NJ) on day 0 or a Revalor-200 on day 0 and again on day 91 (REV-200; 20 mg estradiol + 200 mg TBA; Merck Animal Heath, Madison, NJ).

## Discussion

A shift toward heavier cattle carcasses has led to longer days on feed and, subsequently, more potent implant strategies. The present study compared a potent two-implant strategy, REV-200 on day 0 and again on day 91, with the extended-release implant REV-XH administered on day 0. Considering implant administration induces protein accretion, and Zn is critical in many biological processes that support protein synthesis (Oberleas and Prasad, 1969; Suttle, 2010); the effect of Zn supplemented at national (30 mg/kg DM; NASEM, 2016) or industry (100 mg/kg DM; Samuelson et al., 2016) recommendations was examined.

No difference in final BW between REV-XH and REV-200 heifers was detected in the present study, similar to observations of Ohnoutka et al. (2018) when Revalor-200 was administered on both days 0 and 100 (151, 165, 179, or 193 d on feed). Likewise, when Revalor-XH was compared to a less potent initial implant (Revalor-IH) followed by a Revalor-200 on day 90 (172, 193, or 214 d on feed), nominal differences in performance were observed (Crawford et al., 2018). However, the comparison of Revalor-XS vs. a two-implant strategy of the equivalent hormone potency (Revalor-IS followed by Revalor-S) revealed greater carcass-adjusted final BW and ADG for Revalor-XS steers (Parr et al., 2011). Three additional experiments conducted by Parr et al. (2011) and a study by Nichols et al. (2014) observed no differences in performance using the Revalor-IS/Revalor-S implant program. These studies in combination with the present work indicate little to no difference in performance of cattle receiving an extended-release implant vs. a two-implant strategy of equal or greater hormone potency.

In contrast to many extended-release implant comparison studies (Parr et al., 2011; Nichols et al., 2014; Crawford et al., 2018), BW data from both REV-200 and REV-XH heifers were collected at all interim weigh dates in the present study. The more potent REV-200 treatment exhibited greater ADG during days 0–28 and 91–120, directly following the first and second Revalor-200 implant administration. Furthermore, ADG of REV-XH was numerically greater than REV-200 during days 56–91, presumably due to the coated pellets’ hormonal payout around day 70 as suggested by explant data of a similarly coated implant, Revalor-XR (FDA, 2017b).

Increasing dietary Zn positively affected interim BW during peak implant-induced growth on days 91 (re-implant) and 120. The increase in REV-XH heifer performance between days 56 and 91 corresponds to the release of the second portion of Revalor-XH hormone around day 70. Coinciding with this peak payout of hormone, supplementing IND vs. NRC to REV-XH heifers resulted in 6, 8, and 9 kg advantages on days 91, 120, and 168, respectively. However, in REV-200 heifers, the numerical BW advantage due to IND over NRC is more gradual across the study and peaks at 6 kg on day 120. In alignment with the observed Zn effects in the present study, Huerta et al. (2002) found that steers supplemented with 200 mg Zn/kg DM from ZnSO_4_ had a greater improvement in ADG due to implant than steers fed the basal diet (84 mg Zn/kg DM). Additionally, heifers receiving Revalor-H (140 mg trenbolone acetate + 14 mg estradiol; Merck Animal Health) and supplemented with Zn (75 mg Zn/kg DM) regardless of ZnSO_4_, Zn-methionine, or Zn-propionate source numerically had 6.0% greater ADG compared to un-supplemented heifers consuming a diet containing 52.5 or 50.5 mg Zn/kg DM (Nunnery et al., 2007).

Final BW and carcass characteristics in the present study were nearly identical between IND and NRC for REV-200 heifers. However, IND heifers had greater *longissimus thoracis* muscle fiber diameter than NRC near the end of the study (day 164). Such effects on muscle fiber diameter support the role of Zn in muscle growth. More work is needed to determine the impact of different Zn supplementation strategies on muscle growth in heifers. Furthermore, DMI was minimally influenced by Zn in the present study, similar to the work of others (Greene et al., 1988). Improved performance in IND vs. NRC during the initial implant period resulted in greater G:F during this time, possibly because this period captured greater growth rates from both the hormone release of coated (day 0) and uncoated (day 70) pellets for REV-XH as well as the potent initial Revalor-200 for REV-200 heifers. In the re-implant period, there was a trend for IND to have greater DMI than NRC heifers. This corresponded to a poorer feed efficiency in IND heifers during the terminal implant period, while efficiency was also decreased in REV-XH heifers fed NRC Zn because of poorer gains in this terminal window.

The effect of anabolic implants on trace mineral status of cattle is not well understood. However, the association of Zn with protein synthesis (Oberleas and Prasad, 1969) and Mn with N metabolism as a cofactor for arginase, the terminal enzyme of the urea cycle (Bond et al., 1983; Watts, 1990), suggests that trace minerals are pertinent to implant-induced growth. Administering sheep an implant containing the estrogen derivative zeranol resulted in increased Zn absorption and retention as well as a tendency for increased Mn absorption and retention (Hufstedler and Greene, 1995). Furthermore, concurrent increases in Zn and Mn retention when steers were supplemented 120 mg Zn/kg DM (Carmichael et al., 2018) also indicate a relationship between Zn and Mn utilization in cattle experiencing high growth rates.

Previous work in our laboratory has indicated that administration of a potent terminal implant decreases liver Mn 14-d post-implant (Messersmith, 2018; Niedermayer et al., 2018), and in the present study both implant strategies decreased liver Mn in the first 14 d of the study. The nominal decrease in liver Mn of REV-XH may be due to the lesser potency of hormone released over the 168-d study compared to the REV-200 treatment. Because there were no nonimplanted heifers in this study, it is unclear if REV-XH liver Mn would be lesser than nonimplanted heifers, though this would be anticipated. Increasing hormone potency would be expected to increase cattle growth response (Bartle et al., 1992) and thus muscle catabolism would be decreased in implanted cattle (Galbraith, 1980). We hypothesize that liver Mn is decreased in more potent implant strategies due to lesser demand for the urea cycle and subsequently the Mn-dependent enzyme arginase. Little work has been done to quantify exactly why this change in liver Mn occurs in implanted cattle.

Messersmith (2018) observed a decrease in liver and plasma Zn 14 d following administration of a potent implant (Component TE-200; 200 mg trenbolone acetate + 20 mg estradiol; Elanco Animal Health; Greenfield, IN) compared to nonimplanted steers. A lack of available biomarkers of Zn status complicates the search to better understand the complex interactions between trace minerals and anabolic implant-induced growth. Furthermore, no effect of implants or dietary Zn concentration was found on liver Zn or Cu concentrations, in contrast to work by Niedermayer et al. (2018) who reported liver Cu to be decreased 14 d after delivery of a moderate potency implant.

Consistent with previous work (Bondurant et al., 2018; Ohnoutka et al., 2018), minimal differences in HCW, dressing percent, REA, or YG were observed between Revalor-XH and Revalor-200 followed by a second Revalor-200. Ohnoutka et al. (2018) noted a tendency for Revalor-XH heifers to have better USDA marbling scores than those receiving the Revalor-200 strategy while the current study revealed no differences in marbling due to implant. Overall, the lack of differences in carcass characteristics due to implant strategy suggests that cattle feeders may select whichever strategy best fits their production system. However, Zn supplementation may drive this decision as within REV-XH, heifers fed IND tended to have greater dressing percent than NRC with no dress differences within REV-200. Zinc treatment did not affect additional carcass characteristics in the current study. Effects of Zn supplementation on carcass characteristics have been mixed, likely due to differences in basal Zn concentrations for nonsupplemented animals. Spears and Kegley (2002) observed an increase in quality grade and marbling while both YG and BF tended to increase due to supplementation of 25 mg Zn/kg DM to a basal diet containing either 33 or 26 mg Zn/kg DM for the growing and finishing periods, respectively. Similarly, Malcolm-Callis et al. (2000) observed a quadratic increase in YG and BF due to supplementation of 20, 100, or 200 mg Zn/kg DM from ZnSO_4_ (basal diet ~ 70 mg Zn/kg DM) with steers supplemented 100 mg Zn/kg DM having the greatest BF and YG.

This work suggests that overall performance and carcass characteristics were not drastically affected by either Zn or implant treatment. Revalor-XH is a viable alternative to re-implanting heifers without losing potential gains, though this result appears to be somewhat dependent on dietary Zn concentration. Interim performance improvements due to IND supplementation of Zn provide evidence that Zn is an important trace mineral during periods of high growth rates such as that occurring during peak hormonal payout. Future work is warranted to determine optimal implant strategies for long-fed cattle and refine Zn requirements of cattle receiving anabolic implants to best capture carcass gains.

## Abbreviations

ADG: average daily gain
BF: back fat
BW: body weight
DM: dry matter
DMI: dry matter intake
G:F: gain to feed ratio
HCW: hot carcass weight
KPH: Kidney, pelvic, and heart fat percentage
MGA: melengestrol acetate
PUN: plasma urea nitrogen
REA: ribeye area
TMR: total mixed ration
YG: yield grade
